# Effect of adhesive strategy on the shear bond strength of monochromatic and nanohybrid composite resins to glass ionomer cements in the sandwich technique

**DOI:** 10.3389/fdmed.2026.1728691

**Published:** 2026-02-20

**Authors:** Rizwan Jouhar

**Affiliations:** Department of Restorative Dental Sciences, College of Dentistry, King Faisal University, Al-Ahsa, Saudi Arabia

**Keywords:** composite resin, glass ionomer cement, resin-modified GIC, sandwich technique, shear bond strength

## Abstract

**Background:**

The bilayered (sandwich) technique combines the biological benefits of glass ionomer cements (GICs) with the esthetics and mechanical strength of composite resins. Interfacial bonding in this technique is influenced by GIC type, composite resin, and adhesive strategy. This study evaluated the shear bond strength (SBS) of monochromatic and nanohybrid composite resins bonded to resin-modified (RMGIC) and self-cure GIC using etch-and-rinse and self-etch adhesive protocols.

**Materials and methods:**

Eighty standardized GIC specimens (4 × 3 mm) fabricated from RMGIC and self-cure GIC were randomly assigned to eight groups (*n* = 10). Monochromatic (Vitra Unique) and nanohybrid (Filtek Z250 XT) composite resins were bonded using either an etch-and-rinse (Adper Single Bond 2) or self-etch (Clearfil SE Bond) adhesive. SBS was measured using a universal testing machine, and failure modes were analyzed. Data were evaluated using three-way ANOVA and Tukey's *post-hoc* test (*p* < 0.05).

**Results:**

RMGIC demonstrated significantly higher SBS than self-cure GIC (*p* = 0.006), and the nanohybrid composite resin exhibited higher SBS values than the monochromatic composite resin (*p* = 0.005). A significant interaction between GIC type and adhesive strategy was observed (*p* = 0.033), whereby the etch-and-rinse adhesive enhanced bonding to RMGIC but did not significantly improve bonding to self-cure glass ionomer cement. The highest SBS values were observed when RMGIC was bonded using an etch-and-rinse adhesive and restored with a nanohybrid composite resin, whereas the lowest SBS values occurred when self-cure glass ionomer cement was bonded using a self-etch adhesive and restored with a monochromatic composite resin.

**Conclusion:**

RMGIC combined with an etch-and-rinse adhesive, particularly when restored with a nanohybrid composite, provided superior immediate bond strength compared with self-cure GIC. These results reflect short-term *in vitro* performance and do not represent long-term clinical durability.

## Introduction

The sandwich technique, also referred to as the bilayered or double-laminate technique, is a well-established restorative approach that combines the distinct advantages of glass ionomer cements (GICs) and composite resins (CRs) (1, 2). GICs provide chemical adhesion to tooth structure and fluoride release, which helps prevent secondary caries, while composite resins offer superior esthetics, strength, and wear resistance (3, 4). Despite these advantages, the clinical success of the sandwich technique largely depends on the quality of the bond formed at the GIC–composite interface, which is often the weakest link in the restoration (5).

Resin-modified glass ionomer cements (RMGICs) were developed to overcome the shortcomings of conventional GICs, such as low fracture toughness and brittleness (6). The incorporation of hydrophilic monomers like 2-hydroxyethyl methacrylate (HEMA) into RMGICs improves flexural strength, enhances adhesion to resin-based composites, and facilitates micromechanical and chemical interaction across the interface (7, 8). Conversely, self-cure GICs, though convenient for larger restorations, may have limited cohesive and adhesive potential, which could compromise long-term performance (9). Beyond mechanical considerations, recent in-silico and molecular docking studies have highlighted that resin-based components used in restorative materials may exhibit variable biological interactions at the molecular level, emphasizing the importance of material selection from both mechanical and biocompatibility perspectives (10).

Parallel to these advances, composite resins have evolved significantly. Monochromatic composites, such as Vitra Unique (FGM Dental Group, Brazil), incorporate “smart chromatic technology,” which enables a single shade to adapt to surrounding tooth structure, potentially influencing its bonding interaction with GICs due to its unique resin matrix and optical properties (11). In contrast, nanohybrid composites such as Filtek Z250 XT (3M ESPE, USA) are characterized by high filler content, improved mechanical performance, and excellent wear resistance, making them reliable for posterior restorations (12, 13). While both materials are widely used, limited comparative data exist regarding their performance in the sandwich technique when bonded to different GIC substrates.

Another critical factor influencing interfacial bonding is the adhesive protocol. Total-etch (etch-and-rinse) systems rely on phosphoric acid to increase surface roughness and porosity of the GIC, creating micromechanical retention for resin infiltration (5). Conversely, self-etch systems, which contain acidic functional monomers such as carboxylates or phosphates, simplify application by eliminating the rinsing step while maintaining clinically acceptable bond strengths (14, 15). Although several studies have evaluated bonding between composite resins and glass ionomer cements using the sandwich technique, the findings remain inconsistent. While some studies report superior bonding with etch-and-rinse adhesive systems, others demonstrate comparable or material-dependent outcomes with self-etch adhesives (16–18). These discrepancies are likely related to differences in glass ionomer composition, composite resin formulation, and adhesive strategy, indicating the need for further investigation using contemporary materials.

Similarly, several studies have investigated the bond strength between composite resins and GICs, many have focused on conventional composites or conventional GICs, leaving a gap in the literature regarding newer materials such as Vitra Unique and their performance relative to established nanohybrids like Filtek Z250 XT (19, 20). Moreover, the combined effects of composite type, GIC type, and adhesive strategy have not been comprehensively addressed.

These materials were selected because they represent commonly used contemporary restorative options in clinical practice, allowing clinically relevant comparison of adhesive performance within the sandwich technique. Therefore, this study aimed to evaluate the shear bond strength (SBS) of Vitra Unique and Filtek Z250 XT when bonded to RMGIC and self-cure GIC using total-etch and self-etch adhesive strategies. Understanding the influence of these variables may help optimize restorative protocols and enhance the longevity of sandwich restorations. The null hypothesis was that glass ionomer cement type, composite resin type, and adhesive strategy would not significantly influence the shear bond strength between glass ionomer cement and composite resin.

## Materials and methods

This *in vitro* experimental study evaluated the shear bond strength (SBS) between two composite resins and two glass ionomer cements (GICs) using different adhesive strategies. A total of 80 cylindrical GIC specimens, each measuring 4 mm in diameter and 3 mm in height, were prepared using a standardized Teflon mold. The sample size of 10 per group was determined using OpenEpi software (version 3.01), based on effect sizes reported by Bilgrami et al. (19), who investigated the SBS between resin composites and GIC. With a power of 80% and a significance level of 5%, a minimum of 9 specimens per group was required; therefore, 10 specimens were included in each group to enhance statistical reliability. Two GIC types were evaluated: resin-modified GIC (RMGIC; Riva Light Cure, SDI Limited, Bayswater, VIC, Australia) and self-cure conventional GIC (Riva Self Cure, SDI Limited, Bayswater, VIC, Australia). The restorative materials and adhesive systems used in this study are summarized in Table 1. The materials were manipulated according to the manufacturers’ instructions and placed into molds, covered with Mylar strips, and pressed with glass slides to eliminate voids. RMGIC specimens were light-cured for 20 s at an intensity of 1,400 mW/cm^2^ using an LED curing unit (Starlight Pro, Kulzer), whereas self-cure GIC specimens were allowed to chemically set for 6 min. All specimens were removed from the molds and stored in distilled water at 37 °C for 24 h prior to bonding to simulate initial intraoral maturation.

**Table 1:** Restorative materials and adhesive systems used in the study.

Material category	Material name	Type/description	Manufacturer
Glass ionomer cement	Riva light cure	Resin-modified glass ionomer cement (RMGIC)	SDI limited
	Riva self cure	Conventional glass ionomer cement	SDI limited
Composite resin	Vitra unique	Monochromatic composite resin	FGM dental group
	Filtek Z250 XT	Nanohybrid composite resin	3M ESPE
Adhesive system	Adper single bond 2	Two-step etch-and-rinse adhesive	3M ESPE
	Clearfil SE bond	Two-step self-etch adhesive	Kuraray Noritake Dental Inc.

The bonding surfaces of the GIC specimens were then treated with one of two adhesive strategies. For the etch-and-rinse (total-etch) technique, 37% phosphoric acid gel (Scotchbond Etchant, 3M ESPE, St. Paul, MN, USA) was applied for 15 s, rinsed thoroughly with water for 10 s, and gently air-dried with oil-free compressed air for 5 s to obtain a moist surface. A thin, uniform layer of Adper Single Bond 2 (3M ESPE, St. Paul, MN, USA) was then applied with a microbrush, air-thinned, and light-cured for 10 s. For the self-etch technique, Clearfil SE Bond (Kuraray Noritake Dental Inc., Tokyo, Japan) primer was applied for 20 s, gently air-dried, followed by the application of bonding agent, which was air-thinned and light-cured for 10 s.

**Figure 1:**
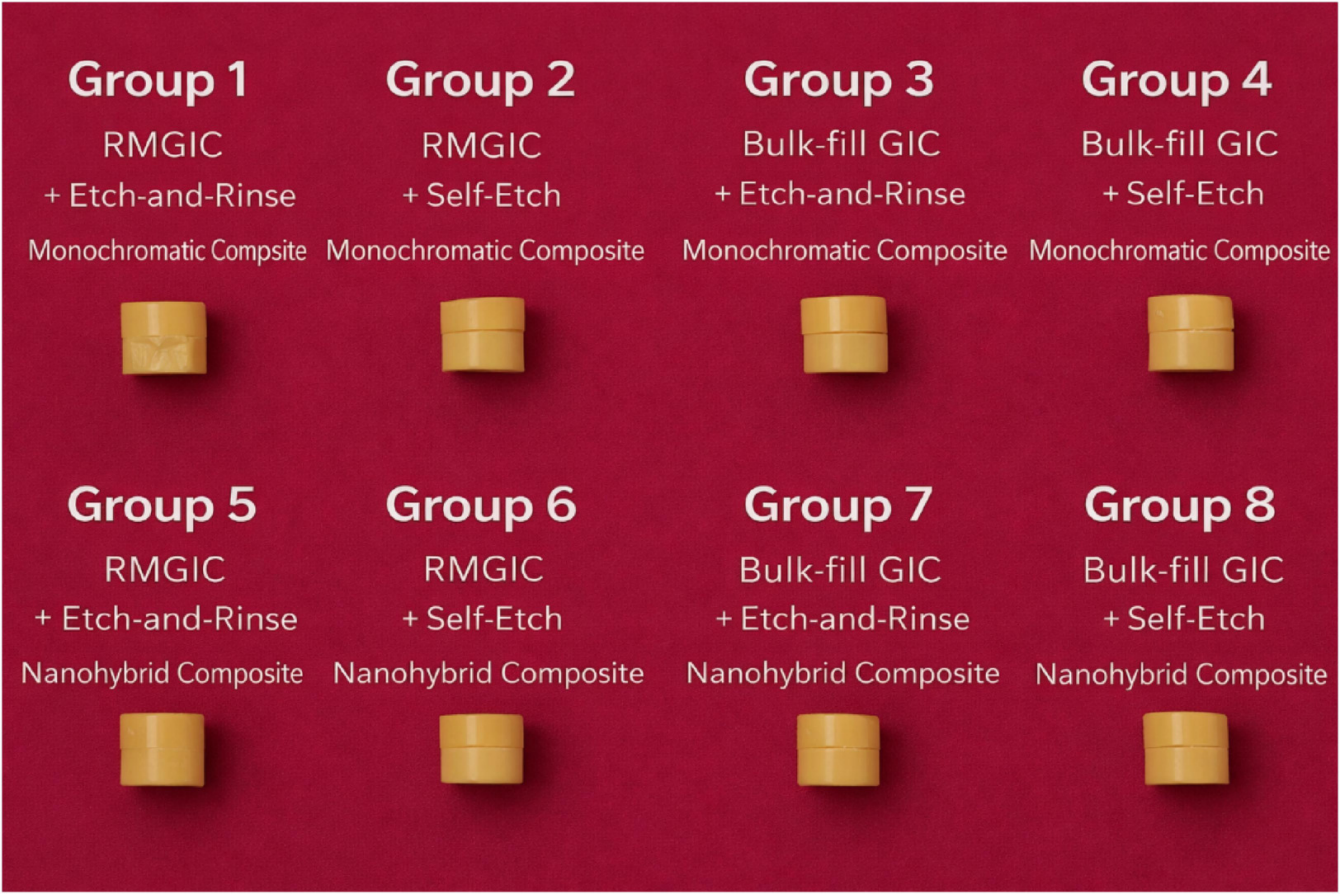
Schematic representation of the experimental grouping based on glass ionomer cement type (resin-modified and self-cure), adhesive strategy (etch-and-rinse and self-etch), and composite resin type (monochromatic and nanohybrid).

Composite build-ups of the same dimensions (4 mm × 3 mm) were placed on the treated GIC surfaces. Two composites were tested: Vitra Unique (FGM Dental Group, Brazil), a monochromatic composite with smart chromatic technology, and Filtek Z250 XT (3M ESPE, St. Paul, MN, USA), a nanohybrid composite with high filler content. Each build-up was placed incrementally in 2 mm layers, with each layer light-cured for 20 s at a distance of 1 mm, using the same LED curing unit (1,400 mW/cm^2^, 440–465 nm wavelength). Based on the type of glass ionomer cement, adhesive strategy, and composite resin, specimens were randomly allocated using a computer-generated random allocation sequence into eight experimental groups (*n* = 10 per group), as illustrated in Figure 1.

To prevent early moisture contamination, specimens were protected with Mylar strips during the initial setting phase and were immersed in distilled water only after completion of the initial setting reaction. Although the sandwich technique is commonly completed in a single clinical session, specimens were stored in distilled water at 37 °C for 24 h following bonding to allow early maturation of the glass ionomer cement and to ensure standardized and reproducible testing conditions prior to shear bond strength evaluation. All specimen preparation and bonding procedures were performed under controlled laboratory conditions at room temperature (23 ± 1 °C) and relative humidity of approximately 50%.

Shear bond strength (SBS) testing was conducted using a Universal Testing Machine (Instron 5965, Canton, MA, USA) with a chisel-shaped loading head positioned perpendicularly to the GIC–composite interface. A crosshead speed of 1 mm/min was applied until failure occurred, and SBS values were recorded in megapascals (MPa). The fractured surfaces were examined under a stereomicroscope (Swift S7-TL, China) at ×20 magnification, and failure modes were classified as adhesive (at the GIC–composite interface), cohesive (within GIC or composite), or mixed. Representative images of each failure mode were recorded for documentation. All experimental procedures and evaluations were carried out by the principal investigator.

### Statistical analysis

Statistical analysis was performed using SPSS version 23.0 (IBM Corp., Armonk, NY, USA). Data normality and homogeneity of variance were verified prior to ANOVA using the Shapiro–Wilk and Levene's tests, respectively. A three-way ANOVA was applied to assess the effects of GIC type, adhesive strategy, and composite resin, as well as their interactions, on SBS values. *post-hoc* pairwise comparisons were performed using Tukey's HSD test. The significance level was set at *p* < 0.05, and effect sizes (partial *η*^2^) were calculated to evaluate the magnitude of each factor.

## Results

**Figure 2:**
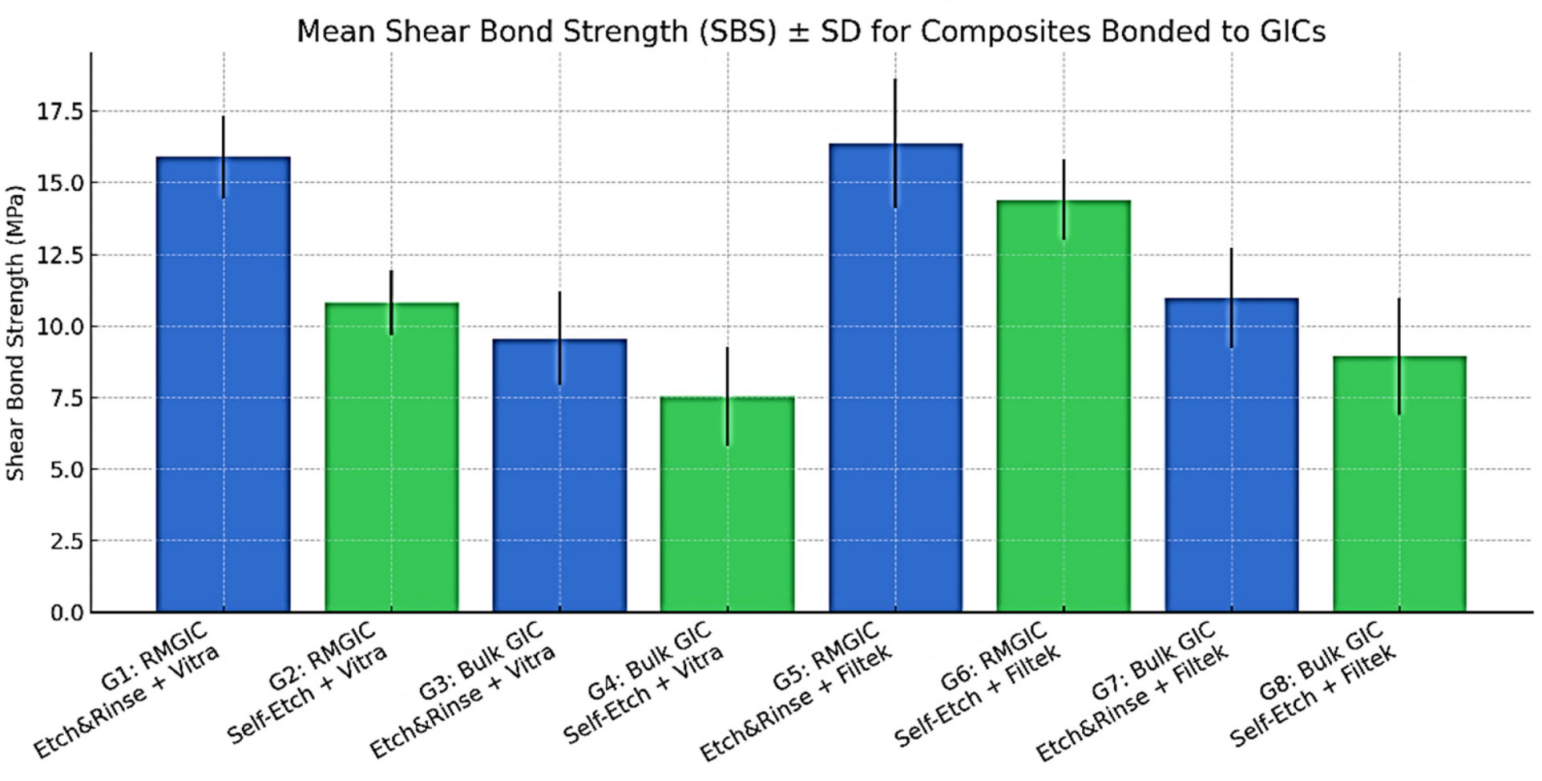
Mean shear bond strength (SBS) values (MPa) with standard deviation (± SD) for monochromatic and nanohybrid composite resins bonded to resin-modified glass ionomer cement (RMGIC) and self-cure glass ionomer cement using etch-and-rinse and self-etch adhesive strategies. Groups involving RMGIC exhibited higher SBS values compared with self-cure glass ionomer cement. Etch-and-rinse adhesive strategy improved bonding performance to RMGIC, particularly when combined with nanohybrid composite resin, whereas self-cure glass ionomer cement showed lower SBS values regardless of adhesive strategy. Error bars represent standard deviations.

A total of 80 specimens were tested across eight experimental groups (*n* = 10 per group). The descriptive statistics (means ± SD) for shear bond strength (SBS) are presented in Figure 2.

Three-way ANOVA revealed significant main effects of GIC type (F = 7.83, *p* = 0.006, partial *η*^2^ = 0.12) and composite type (F = 8.37, *p* = 0.005, partial *η*^2^ = 0.13), indicating that RMGIC exhibited higher SBS than self-cure GIC, and Filtek Z250 XT produced higher SBS than Vitra Unique. In contrast, the adhesive strategy alone did not have a significant main effect on SBS (F = 0.08, *p* = 0.782).

Importantly, a significant GIC × Adhesive interaction was detected (F = 4.67, *p* = 0.033, partial *η*^2^ = 0.08), indicating that the effectiveness of the adhesive system depended on the type of GIC. Specifically, total-etch (etch-and-rinse) adhesives significantly enhanced the bonding of RMGIC compared to self-etch, whereas self-cure GIC exhibited consistently low SBS regardless of the adhesive used. Neither the GIC × Composite (F = 0.03, *p* = 0.852) nor the Adhesive × Composite (F = 1.25, *p* = 0.265) interactions were statistically significant. The three-way interaction approached significance (F = 3.29, *p* = 0.072), suggesting a possible combined effect of material and adhesive selection (Table 2).

**Table 2:** Three-way ANOVA results for the effects of GIC type, adhesive strategy, and composite type on shear bond strength (SBS).

Variation	df	F-value	*p*-value	Partial *η*^2^
GIC type (RMGIC vs. self-cure)	1	7.83	0.006**	0.12
Adhesive strategy (etch vs. self-etch)	1	0.08	0.782 (NS)	0.00
Composite type (Vitra vs. Filtek)	1	8.37	0.005**	0.13
GIC × adhesive	1	4.67	0.033*	0.08
GIC × composite	1	0.03	0.852 (NS)	0.00
Adhesive × composite	1	1.25	0.265 (NS)	0.02
GIC × adhesive × composite	1	3.29	0.072 (NS)	0.05

NS, not significant.

* *p* < 0.05.

** *p* < 0.01.

**Figure 3:**
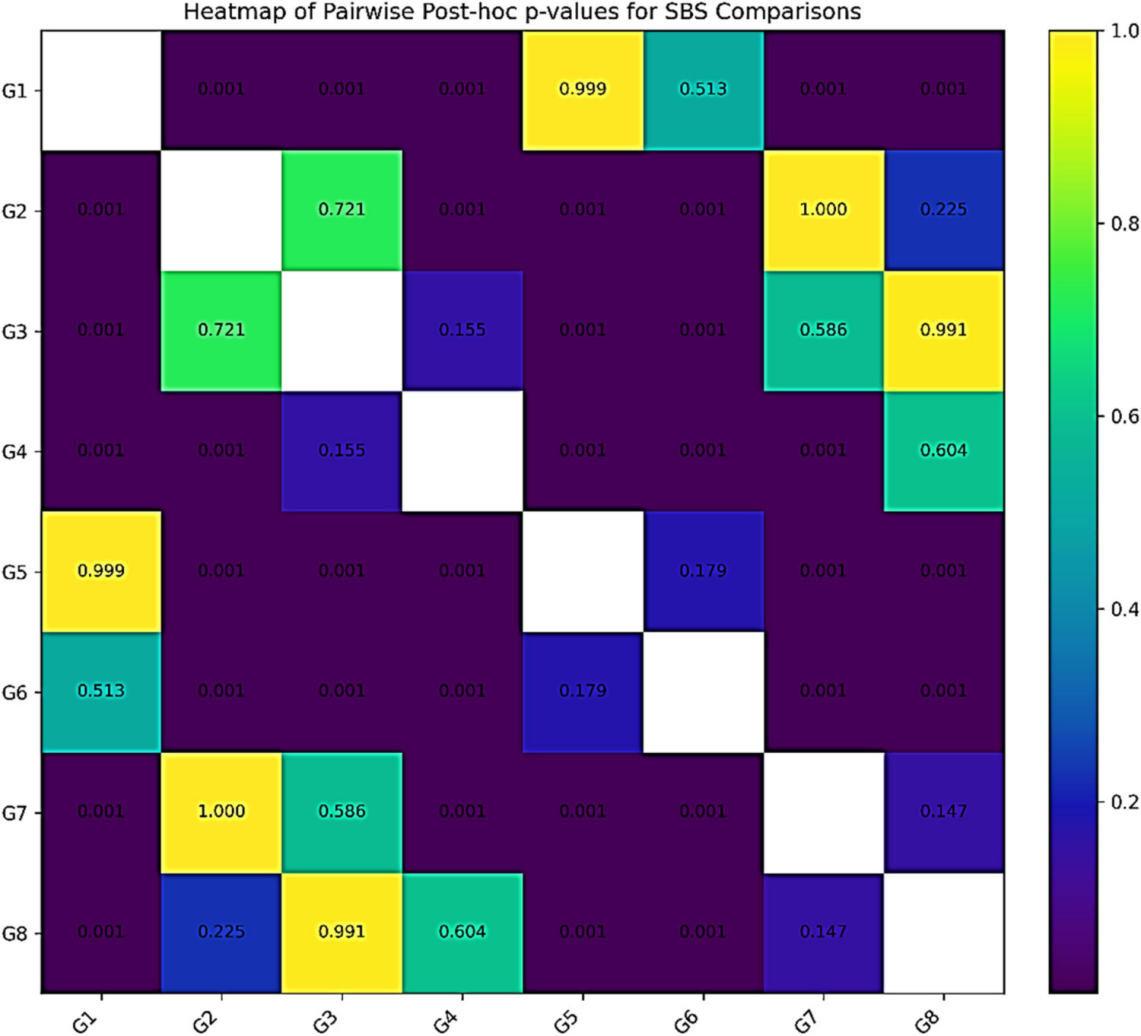
Heatmap illustrating pairwise *post-hoc* comparisons of shear bond strength (SBS) among the eight experimental groups based on tukey's test following three-way ANOVA. Each cell displays the corresponding *p*-value for the comparison between two groups. Darker shades represent stronger statistical differences, whereas lighter shades indicate non-significant differences (*p* ≥ 0.05).

Post-hoc Tukey's HSD comparisons supported these findings, with pairwise group differences visually summarized in a heatmap as shown in Figure 3. The highest SBS was recorded for Group 5 (RMGIC + Etch-and-Rinse + Filtek Z250 XT: 16.37 ± 2.25 MPa), which was significantly greater than all self-cure GIC groups (*p* < 0.001), but not significantly different from Group 1 (RMGIC + Etch-and-Rinse + Vitra Unique: 15.90 ± 1.45 MPa, *p* = 0.987) or Group 6 (RMGIC + Self-Etch + Filtek Z250 XT: 14.40 ± 1.41 MPa, *p* = 0.448). Similarly, Group 1 showed significantly higher SBS than Groups 2, 3, 4, 7, and 8 (all *p* < 0.001), but not from Groups 5 or 6. The lowest SBS was observed in Group 4 (Self-cure GIC + Self-Etch + Vitra Unique: 7.54 ± 1.72 MPa), which was significantly lower than all RMGIC groups (*p* < 0.001). Overall, RMGIC bonded with total-etch adhesives consistently outperformed self-cure GIC, while Filtek Z250 XT showed superior performance compared to Vitra Unique, though the difference was not always statistically significant at the group level.

**Figure 4:**
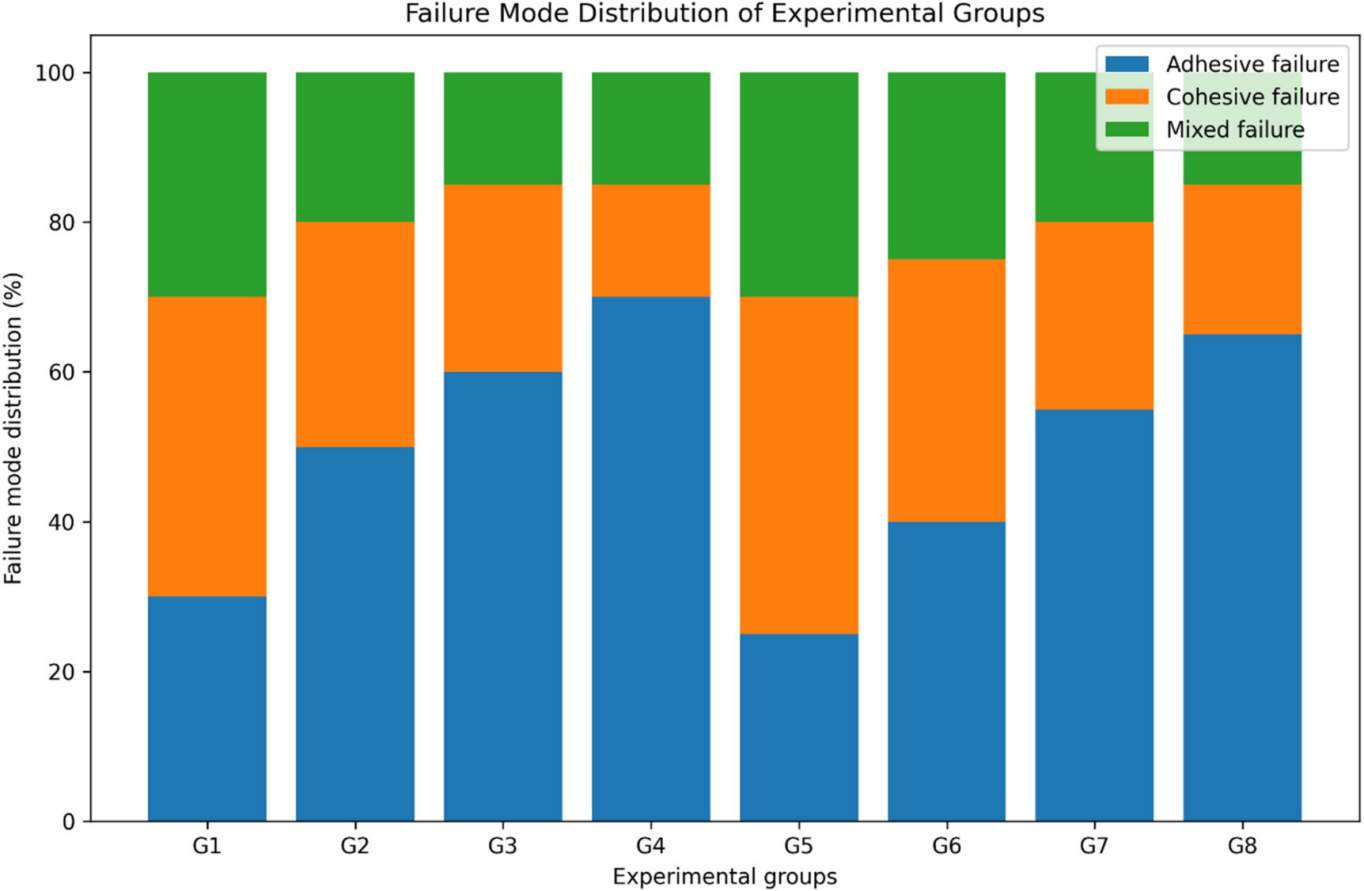
Stacked bar chart illustrating the distribution of adhesive, cohesive, and mixed failure modes (%) observed across the eight experimental groups following shear bond strength testing. Groups exhibiting higher bond strength demonstrated a greater proportion of cohesive failures, whereas groups with lower bond strength predominantly showed adhesive failures.

**Figure 5:**
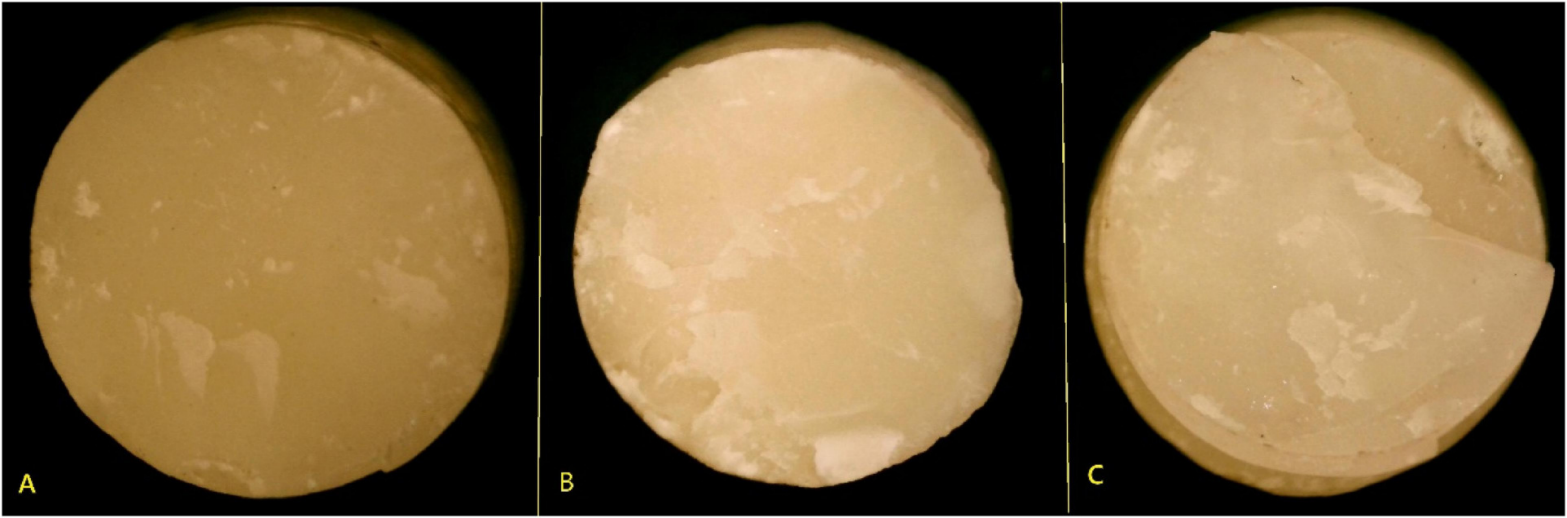
Representative images of failure modes: **(A)** adhesive, **(B)** cohesive, and **(C)** mixed failures observed post-bond strength testing.

Failure mode analysis confirmed the SBS results (Figures 4, 5). Adhesive failures were most frequent in self-cure GIC with self-etch adhesives, particularly Group 4 (70%) and Group 8 (65%), consistent with their lower SBS values. In contrast, cohesive failures were more frequent in RMGIC with etch-and-rinse adhesives, especially in Group 5 (45%) and Group 1 (40%), suggesting stronger internal integrity. Mixed failures were observed across groups, ranging from 15% to 30%, and were most frequent in the high-strength groups (Groups 1 and 5, 30%).

Overall, RMGIC combined with etch-and-rinse adhesive demonstrated superior shear bond strength compared with self-cure GIC, particularly when restored with nanohybrid composite resin. These findings highlight the influence of material selection and adhesive strategy on immediate interfacial bonding.

## Discussion

The sandwich technique is widely recognized for restoring deep cavities by combining the favorable biological properties of glass ionomer cements (GICs) with the aesthetics and mechanical performance of composite resins (CRs) (1, 21). The success of this approach depends largely on the quality of adhesion between the two materials. In the present study, the shear bond strength (SBS) of two composites, Vitra Unique and Filtek Z250 XT, bonded to two GICs, resin-modified GIC (RMGIC) and self-cure GIC, using etch-and-rinse or self-etch adhesives, was investigated.

The results demonstrated that GIC type significantly influenced SBS, with RMGIC consistently outperforming self-cure GIC (*p* = 0.006). This finding is consistent with previous studies reporting that the incorporation of resin monomers such as HEMA improves the mechanical properties and handling characteristics of RMGICs, which may indirectly contribute to improved bonding performance when compared with conventional GICs (2, 3). HEMA promotes better infiltration and covalent bonding through unreacted methacrylate groups, improving both chemical and micromechanical adhesion (9, 18). Previous studies have demonstrated that modifications to RMGIC formulations, including filler optimization, can enhance mechanical stability, which may influence overall bond strength measurements without necessarily indicating improved interfacial adhesion (22). In contrast, self-cure GICs exhibited lower SBS values, which may be related to differences in material composition and surface characteristics that affect interaction with adhesive systems, rather than cohesive strength alone (12, 23). These findings underscore the material-specific behavior of glass ionomer cements under different conditions. Previous studies have also shown that external factors, such as exposure to static magnetic fields, can influence the mechanical properties and integrity of glass ionomer cements, further highlighting the material-dependent nature of their performance (24, 25).

Composite type also had a significant effect (*p* = 0.005), with Filtek Z250 XT showing superior SBS compared with Vitra Unique. The higher SBS observed with Filtek Z250 XT may be associated with differences in composite resin formulation and interaction with the adhesive layer, rather than mechanical properties alone (10). Vitra Unique, although clinically appealing for its smart chromatic technology, demonstrated lower shear bond strength values, particularly when bonded to self-cure glass ionomer cement and when used in combination with self-etch adhesive systems. This reduced bonding performance may be related to differences in resin matrix composition and filler characteristics compared with nanohybrid composites, which can influence interaction with the adhesive layer. Previous studies have shown that variations in composite resin chemistry can affect bonding behavior to resin-modified glass ionomer cements, even when similar adhesive protocols are used (26). Consistent with this, earlier investigations have reported superior bonding performance of nanohybrid composites compared with certain experimental or shade-adaptive resin materials (13). However, the exact mechanisms underlying these differences were not investigated in the present study.

Although the adhesive strategy alone did not significantly influence shear bond strength (*p* = 0.782), a significant interaction between adhesive strategy and glass ionomer cement type was observed (*p* = 0.033). Etch-and-rinse adhesives enhanced bonding performance when used with resin-modified glass ionomer cement (RMGIC), whereas self-cure glass ionomer cement exhibited consistently low bond strength irrespective of the adhesive system applied. Etch-and-rinse adhesives may enhance bonding to RMGIC by modifying the cement surface and increasing surface roughness, thereby facilitating improved interaction with the adhesive resin; however, no microscopic evaluation was performed in this study to directly confirm these surface changes. Previous literature has emphasized that adhesive systems do not perform uniformly across different substrates and material compositions, highlighting the substrate-dependent nature of adhesive performance (27). In contrast, excessive acid conditioning of conventional glass ionomer cements has been reported to adversely affect their structure by dissolving surface components and weakening cohesive integrity, which may explain the limited benefit of etch-and-rinse strategies observed with self-cure glass ionomer cement (28). These findings underscore the importance of selecting adhesive protocols according to the specific restorative substrate.

Failure mode analysis provided additional insight into these findings. Adhesive failures predominated in self-cure GIC groups, particularly those bonded with self-etch adhesives (Groups 4 and 8), reflecting weaker interfacial bonding. In contrast, cohesive and mixed failures were more frequent in RMGIC groups treated with etch-and-rinse adhesives (Groups 1 and 5), suggesting that the interfacial bond strength exceeded the cohesive strength of the GIC itself. This is in line with previous studies reporting that cohesive failures in RMGIC indicate improved internal integrity due to resin reinforcement (29, 30). Bilgrami et al. (19) similarly demonstrated that resin composites bonded to conventional GIC showed variable SBS but tended to fail adhesively, whereas resin-modified materials showed improved interfacial performance. Comparisons with calcium silicate–based materials such as Biodentine and Theracal LC also revealed lower bond strengths than RMGIC, further validating its superior bonding performance (31).

From a clinical standpoint, the results of this study highlight that the most favorable combination for sandwich restorations was RMGIC bonded with an etch-and-rinse adhesive and restored with a nanohybrid composite such as Filtek Z250 XT. This combination not only provided the highest SBS values but also demonstrated more cohesive failures, reinforcing its clinical durability. On the other hand, the use of self-cure GIC, especially with self-etch adhesives, produced the weakest performance and may compromise the long-term success of restorations. Moreover, the durability of self-adhesive systems remains questionable in the long term compared to conventional approaches, which underlines the clinical importance of adhesive selection (32).

Despite these encouraging findings, some limitations must be acknowledged. As an *in vitro* study, it does not replicate intraoral conditions such as variations in pH, saliva composition, occlusal stress, or thermal cycling. The sample size was modest (*n* = 10 per group), and only two adhesive systems and two types of GICs and composites were tested. Future studies should employ larger sample sizes, artificial aging protocols such as thermocycling and cyclic loading, and incorporate novel adhesives, including universal and bioactive materials, to better simulate clinical conditions and validate these results.

From a clinical perspective, the findings suggest that material selection and adhesive strategy play a critical role in the immediate bonding performance of sandwich restorations. The use of resin-modified glass ionomer cement in combination with an etch-and-rinse adhesive and a nanohybrid composite may provide more favorable interfacial bonding compared with self-cure glass ionomer cement, particularly when self-etch adhesives are used. These results may assist clinicians in selecting restorative combinations that optimize immediate bond strength in deep cavity restorations.

## Conclusion

RMGIC combined with an etch-and-rinse adhesive demonstrated superior shear bond strength compared with self-cure GIC, particularly when paired with Filtek Z250 XT. Self-cure GIC with self-etch adhesive showed the weakest performance, underscoring the importance of selecting the appropriate GIC, composite resin, and bonding strategy to optimize the sandwich technique. These findings are limited to immediate bond strength and do not reflect long-term durability. Based on the present findings, clinicians may consider using resin-modified glass ionomer cement in combination with an etch-and-rinse adhesive and a nanohybrid composite when performing sandwich restorations in deep cavities to achieve more favorable immediate bonding performance.

## Data Availability

The raw data supporting the conclusions of this article will be made available by the author, without undue reservation.
